# Occipital nerve stimulation in medically intractable chronic cluster headache

**DOI:** 10.1111/ene.16212

**Published:** 2024-01-17

**Authors:** Casper S. Lansbergen, Cecile C. de Vos, Roemer B. Brandt, Michel D. Ferrari, Frank J. P. M. Huygen, Rolf Fronczek

**Affiliations:** ^1^ Department of Anaesthesiology Erasmus Medical Center Rotterdam the Netherlands; ^2^ Department of Neurology Leiden University Medical Center Leiden the Netherlands

## CONFLICT OF INTEREST STATEMENT

None of the authors has any conflict of interest to disclose.

Dear Editor,

We read with great interest the recently published European Academy of Neurology (EAN) guidelines for the treatment of cluster headache [1]. We applaud the careful and comprehensive analysis of the various acute and prophylactic treatment options. However, we were surprised by the recommendation to use sphenopalatine ganglion stimulation (SPG) and not occipital neurostimulation (ONS) as last resort prophylactic therapy in medically intractable chronic cluster headache (MICCH).

To our knowledge, SPG is no longer available and is not formally approved in any country for the treatment of cluster headache. Although it is effective in the acute treatment of cluster headache attacks [2, 3], there is no randomized controlled evidence for prophylactic efficacy. A potential preventive effect was observed in an uncontrolled open‐label extension study, and suggested in a double‐blind randomized controlled trail on the acute effect of SPG on cluster headache attacks, not the preventive effect [2, 3].

In contrast, ONS was found to be rapidly and long‐term (>5 years) prophylactically effective (50% reduction of attack frequency), safe, and well tolerated in the randomised controlled ICON trial [4] and the recently published prospective long‐term follow‐up L‐ICON study [5]. The proportion of satisfied patients was also extremely high (>90%). These positive findings were reaffirmed in a systematic review and meta‐analysis also published after the EAN taskforce literature review [6, 7]. ONS is now officially approved and reimbursed in several European countries for the prophylactic treatment of MICCH.

The EAN guideline raised concerns about a poor safety profile of ONS based on studies conducted in the early stages of ONS development [1]. Hardware‐related adverse events (e.g., lead migration or battery depletion) did occur in the early phase of the ICON trial. These were mainly due to short battery life and (off‐label) suboptimal leads that were originally intended for use in spinal cord stimulation and were less suitable for placement on the back of the head, where more flexibility is required. Recent developments (e.g., improved implantation procedures, better anchored flexible leads better suited for placement at the back of the head, and less need to replace batteries) have significantly reduced the occurrence of such hardware‐related adverse events. In the prospective long‐term ONS follow‐up L‐ICON study, these improved techniques resulted in a hardware‐related serious adverse event rate of 0.35 per person‐year [95% CI 0.28 – 0.41]. No biological complications (e.g., serious wound infection) were reported [5].

ONS also proved to be cost‐effective compared to conventional therapy in the treatment of MICCH, with an average lower annual cost of €1344 [8]. This study also observed a significant improvement in quality of life, with a gain of 0.28 quality‐adjusted life years. In the Netherlands, we now successfully implant ONS in approximately 60 MICCH patients per year, with hardly any safety or tolerability problems.

In conclusion, SPG has not (yet) been shown to have a prophylactic effect in cluster headache and is no longer available. In contrast, ONS has proven to be an effective, well‐tolerated, and safe treatment option for patients with chronic cluster headache who do not respond to standard medical treatment. It is also cost‐effective and accepted as reimbursed care by several European countries. Ongoing developments in hardware and implantation procedures ensure ever lower risk of complications. We realize that some of the above information became available only after the EAN task force's literature review and could not be included in the guidelines, yet urge that the EAN guideline be updated accordingly.

## AUTHOR CONTRIBUTIONS

**Casper S. Lansbergen:** Writing – original draft; writing – review and editing. **Cecile C. de Vos:** Supervision; writing – review and editing. **Roemer B. Brandt:** Supervision. **Michel D. Ferrari:** Supervision; writing – original draft; writing – review and editing. **Frank J. P. M. Huygen:** Supervision. **Rolf Fronczek:** Writing – review and editing; supervision; conceptualization.

## Data Availability

Data sharing not applicable to this article as no datasets were generated or analysed during the current study.
